# Whole School SEND (WSS) Review: study protocol for a two-arm pragmatic parallel cluster randomised controlled trial in 160 English secondary schools

**DOI:** 10.1186/s13063-021-05280-y

**Published:** 2021-05-10

**Authors:** Stephen Morris, Andrew Smith, Cathy Lewin, Peter Hick, Jordan Harrison

**Affiliations:** 1 Policy Evaluation and Research Unit, Manchester Metropolitan University, Manchester, UK; 2 Education and Social Research Institute, Manchester Metropolitan University, Manchester, UK; 3 School of Childhood, Youth & Education Studies, Manchester Metropolitan University, Manchester, UK

**Keywords:** Cluster randomised, Special educational needs, Attainment, Wellbeing, Schools

## Abstract

**Background:**

The trial will study the effects of the Whole School SEND Review on secondary school pupils in English mainstream education, to understand the impact of the intervention on academic attainment, wellbeing, and school attendance. The Review is designed to facilitate whole-school change through providing enhanced, intensive and sustained support and training in inclusive education for school special educational needs coordinators and leadership teams. The trial will have a specific focus on pupils designated as having special educational needs or disabilities.

**Methods:**

We recruited 160 English secondary schools (approx. 58,000 pupils across two cohorts) to a two-arm pragmatic parallel cluster randomised controlled trial, with allocation at the school level. Randomisation will be stratified by school region. The primary outcome is attainment in English language (using standardised national test results at 16 years) for pupils designated as having a special educational need (approx. 4000 pupils). Secondary outcomes will be measured for pupils both with and without a special educational need designation and include pupil wellbeing (measured using the Strengths and Difficulties Questionnaire), absences and exclusions, and attainment in Mathematics and English language at 16 years. The intervention will be implemented from July 2021 and analysis of outcomes (for the year 9 cohort) will take place in September 2023, with further analysis (for the year 8 cohort) in September 2024 if the evaluation shows that acceptable implementation fidelity has been achieved.

**Discussion:**

Pupils with special educational needs represent a significant and often vulnerable part of the secondary school population, are disproportionately likely to be excluded from school, eligible for free school meals, or supported by children’s social care. Despite these multiple important areas of need, school leaders report substantial challenges in making additional provision for this group. Previous research has highlighted the development of inclusive school cultures (rather focusing primarily on targeted individualised approaches) as being important. This trial will investigate how an intervention designed to drive whole school change may lead to outcomes for pupils with and without a special educational needs designation. As such, this trial is expected to make an important contribution to research evidence and to UK educational policy.

**Trial registration:**

ISRCTN registry ISRCTN11339306. Registered on 12 March 2020 (retrospectively registered).

## Administrative information

**Table:** 

Title {1}	Whole School SEND (WSS) Review: study protocol for a two-arm pragmatic parallel cluster randomised controlled trial in 160 English secondary schools
Trial registration {2a and 2b}.	This trial is registered at the ISRCTN registry, registration number ISRCTN11339306 (ISRCTN, 2020) [1]
Protocol version {3}	Version 2.0 (30^th^ October 2020). Revised protocol accounting for changes stemming from school closures in response to Covid-19 pandemic (timelines further subject to minor amendments in this document).
Funding {4}	The Education Endowment Foundation (EEF) has granted £458,699 to fund the evaluation. The EEF had no direct role in the design of this study, although they were consulted throughout the design stage. During execution, their role will be limited to helping to advertise the study (to aid recruitment). They will have no role in the analyses or interpretation of the data.
Author details {5a}	Stephen Morris, Professor of Evaluation^a^ Andrew Smith, Research Associate^a^ Cathy Lewin, Professor of Education^b^ Dr Peter Hick, Principal Lecturer, Inclusive Education^c^ Jordan Harrison, Senior Research Assistant^a^ ^a^Manchester Metropolitan University, Policy Evaluation and Research Unit ^b^Manchester Metropolitan University, Education and Social Research Institute ^c^Manchester Metropolitan University, School of Childhood, Youth & Education Studies
Name and contact information for the trial sponsor {5b}	Jennifer Stevenson, Evaluation Manager The Education Endowment Foundation 5^th^ Floor, Millbank Tower 21-24 Millbank London SW1P 4QP
Role of sponsor {5c}	See item 4 (Funding) above.

## Introduction

### Background and rationale {6a}

The trial will study the effects of the Whole School Send (WSS) Review on secondary school pupils in English mainstream education to answer research questions relating to academic attainment, school attendance, and wellbeing. The intervention has been developed by the National Association for Special Educational Needs (nasen), and the evaluation is being sponsored and funded by the Education Endowment Foundation (EEF). Manchester Metropolitan University have been appointed as researchers.

A group of pupils of particular interest are pupils with special educational needs or disabilities (SEND) who represent a significant and often vulnerable part of the secondary school population (14.9%) [2]. School leaders report substantial challenges in making additional provision for SEND pupils, who are disproportionately likely to be excluded from school, to be eligible for free school meals and to be ‘looked after’ or identified as a child in need [3].

Research over recent years has highlighted the importance of inclusive pedagogy for all learners [4–6], pointing to the need to develop schools as inclusive learning environments, rather than focusing primarily on specialist approaches for individuals identified with SEND. Equally, there is a strand of research in the field of inclusive education over the last thirty years addressing the development of more inclusive practices with learners with SEND, as a whole-school development issue [7]. A key example is the Index for Inclusion [8], which provides a process and resources to support inclusive school development.

The WSS Review is best understood against this backdrop of wider research examining inclusive school cultures. The WSS Review process includes the following:

- SEND Co-ordinator (SENDCo) training on reviewing and peer mentoring provided by an experienced SEND reviewer.
- The use of an evidence-based framework which draws on a school’s current information, robust data and contextual factors to structure the review.
- Peer-to-peer support and a reflection network to facilitate a collaborative, localised and grassroots approach to developing SEND provision.

The process reflects the premise that excellent teaching for pupils with SEND is excellent teaching for all, but there is a dearth of rigorous evaluation evidence relating to specific whole-school level interventions that are relevant to secondary schools and can be adopted at scale.

The WSS Review was developed by nasen in response to the Department for Education (DfE) identifying a need for schools to access support for implementing 2014 Special Educational Needs and Disability (SEND) reforms [9]. DfE guidance encourages schools to commission a review using the WSS materials, to reflect on SEND provision and explore different approaches to raising attainment.

An evaluation of the initial DfE contract for WSS delivery^9^ noted evidence of promise in terms of the impact at the school level, for example:

- The process enabled schools to build on what they were already doing well for pupils.
- More non-specialist SEND teachers were willing to look reflectively at their classroom practice.
- Subject leaders became more aware of SEND practice and curriculum differentiation.
- A wider awareness of the value of pupil progress data and its use in future curriculum planning was developed.
- SEND operational practices were changed in some schools.
- Peer-to-peer mentoring was adopted by some teachers/groups outside of SEND.
- The use of peer-to-peer mentoring was valued by all participants.

However, the evaluation noted that there were particular challenges with the review process in secondary school settings, due to the size of such institutions resulting in more children designated as SEND and the greater chance of inconsistencies in practices and staff attitudes to SEND provision, as well as the behaviour of SEND teams working independently of each other [9]. A further additional challenge relates to the tension between secondary schools working together and the competition between them in relation to student recruitment.

The evaluation did not specifically evaluate the effects of the WSS Review on pupil attainment, attendance and wellbeing, and this is therefore a logical next step, particularly given the anticipated impacts of the WSS Review on pupils. Furthermore, the WSS Review includes a specific focus on learners with identified SEND whereas other initiatives have tended to give greater emphasis to the need to move beyond SEND to a broader approach to include all learners. This trial will therefore investigate the outcomes for pupils with and without a SEND designation.

### Objectives {7}

The primary objective of this trial is to test the efficacy of the WSS Review at *raising attainment in English Language at General Certificate of Standard Education (GCSE)* among pupils designated SEND. Other objectives are to test the efficacy of the WSS Review (for pupils both with and without a SEND designation) at:

- Raising attainment in GCSE mathematics
- Reducing absences and exclusions from school
- Improving pupils’ wellbeing

The trial is therefore designed to test the WSS Review theory of change (see the ‘Discussion’ section), which assumes the widespread effects of the intervention towards transformational change both for pupils with and without a SEND designation. It is expected that this to occur through fostering and promoting a more inclusive and supportive school culture and that the intervention will have a direct effect on the classroom through encouraging SENDCos to engage more fully with both teaching practice and the learning environment. We therefore also expect a consequent improvement in pupils’ attainment, particularly among pupils designated SEND. The WSS Review is hypothesised to enhance the inclusivity and supportiveness of the school, and as such is anticipated to reduce absences and exclusions particularly among pupils designated SEND. It is also expected that the Review will improve pupils’ wellbeing through similar mechanisms.

### Trial design {8}

To test these hypotheses, we will conduct a two-arm pragmatic parallel cluster randomised controlled trial with allocation at the school level (allocation ratio 1:1). This involves the recruitment of a sample of schools from across England that are subsequently assigned at random to intervention and control groups; the intervention schools receiving the intervention, the control school prohibited from doing so.

Schools are chosen as the unit or level at which randomisation occurs because of the ‘whole-school’ nature of the intervention. This feature of the intervention makes the randomisation of individual pupils or whole classes infeasible. Randomisation of schools and the measurement of outcomes at the pupil level implies a multi-level or hierarchical data structure, with pupils clustered within classes and classes with schools.

Randomisation will be stratified by region in order to facilitate regional recruitment and training, to avoid unnecessary delay, and to achieve balance in the number of schools within intervention and control arms within each region.

## Methods: participants, interventions and outcomes

### Study setting {9}

Data will be collected from mainstream secondary schools (for pupils aged 11–16 years) in England in five regions (see the ‘Eligibility criteria {10}’ below). A list of study sites will not be publicly available until the trial is completed, at which point it will be deposited in the EEF data archive in the Office for National Statistics (ONS) Secure Research Service (SRS).

### Eligibility criteria {10}

Inclusion criteria have been applied at the level of both the school and pupil. Schools with the following criteria were eligible for recruitment to the study sample, such that the school:

- Is a mainstream secondary school.
- Must not have previously commissioned a WSS Review.
- Must be located in one of the following regions of England: North East, North West, South Central England and North West London, South West and West Midlands.
- SENDCo and other members of the school leadership team have not previously engaged with the WSS Review or similar audit.

Only one school per multi-academy trust (MAT) qualifies for inclusion in the trial. This is because MATs often set policy in relation to SEND centrally and we wish to avoid a situation where schools from the same MAT are assigned to intervention and control groups.

Within each eligible school the following pupils meet the inclusion criteria for this trial:

- All pupils in years 8 and 9 on Tuesday 1st September 2020

### Who will take informed consent? {26a}

In order to participate in the trial, schools were required to sign a Memorandum of Understanding (MoU), which sets out their obligations as well as those of the developers and evaluators. The MoU is signed by the chair of school governors, the head teacher and the school SENDCo. Parents have been issued with a Parent Information Sheet outlining their child's involvement in the project, a Privacy Notice outlining how their child’s data will be managed and a Withdrawal Form [10]. Parents have been encouraged to discuss the project with their child before deciding whether or not to complete the Withdrawal Form.

### Additional consent provisions for collection and use of participant data and biological specimens {26b}

This trial does not involve collecting biological specimens.

## Interventions

### Explanation for the choice of comparators {6b}

Schools allocated to the control group will operate under ‘business as usual’; that is, they will not participate in the WSS Review process during the trial, but will be able to access it from September 2024. They may however decide to develop SEND provision by accessing alternative resources during the trial period. The Implementation Process Evaluation (IPE) running alongside the trial (see the ‘Discussion’ section) will gather data about this, so that this issue can be considered when interpreting results from the trial.

Schools in the control arm will receive a financial payment of £1500 in two instalments (July 2022, July 2023), on completion of the follow-up administration of the Strengths and Difficulties Questionnaire (SDQ; see the ‘Outcomes {12}’ section below).

### Intervention description {11a}

The underlying model of the WSS Review is peer-to-peer support facilitated by partnering schools (intervention group schools will be partnered with one another). The intervention is delivered to SENDCos who are expected to oversee the Whole School SEND (WSS) Review within their own school and to develop and implement a SEND Development Plan, targeting areas for improvement. Partner SENDCos also engage in peer-to-peer review of each other’s SEND provision, and mutual support throughout the trial.

The WSS Review process aims to raise awareness and give SENDCos more status such that they can become agents of change. Their role should shift from one with a pastoral focus to one that drives change in both teaching and learning. School partnering is pragmatic, being primarily based on geographic proximity, although other considerations such as advice from the regional nasen co-ordinator will also contribute to decisions that are made.

The programme is structured around five key contacts between nasen and the school/SENDCos, which provide a clear framework for ongoing support for each school, alongside a community of practice:

1. SEND Reviewer training (in two parts, July 2021 and September 2021)
2. First engagement day (October 2021)
3. First support visit from WSS project director (October 2021–January 2022)
4. Second engagement day (February–March 2022)
5. Second support visit from WSS project director (March–June 2022)

The *SEND Reviewer training* is a 1-day face-to-face event for SENDCos, facilitated by WSS Project Directors (nasen) to outline the project vision and aims and to deliver SEND Reviewer and peer mentor training. SENDCos from partner schools will jointly attend a training session in their region and training will be delivered to a standardised plan.

Between the training and the first engagement day, partner schools will do a peer-to-peer review of each other’s SEND provision in accordance with a structured process. Doing so will enable them to identify strengths, areas for development and stakeholders who might be involved in initiatives to support SEND students.

The *first engagement day* will be held on a regional basis and will provide an opportunity for SENDCos to begin to write their school’s SEND Development Plan, specifying three areas for development, relevant actions for each area and key stakeholders to involve. The engagement day will also form the basis of a regional community of practice for sharing knowledge, ideas and experience. It will also provide opportunities for collaborating and developing local networks.

The draft Development Plan will be shared with senior leaders and governors at a Full Governing Body meeting before the *first support visit by a WSS Project Director.* The purpose of the visit will be to review the school’s SEND Development Plan with the SENDCo and a senior leader, as well as to meet the head teacher, provide one-to-one coaching and address the SENDCo-led agenda for the day. Following the first support visit, the SENDCo will work with senior leaders to implement the SEND Development Plan.

A *second engagement day* will provide tailored SEND development based on regional feedback from school visits, review the engagement of stakeholders and facilitate regional sharing of best practice. Following this event, SENDCos will continue to work with senior leaders to implement their schools’ SEND Development Plan.

Finally, a *second support visit by a WSS Project Director* will discuss the action plan implementation, reviewing progress and next steps for SEND provision, providing a one-to-one coaching session and collecting anecdotal evidence to support a final review report.

### Criteria for discontinuing or modifying allocated interventions {11b}

Less confident SENDCos (ascertained at the first engagement day) will be visited first to ensure that they are better placed to implement their plans straight away.

### Strategies to improve adherence to interventions {11c}

The project team has established a strong communications strategy that emphasises the importance of schools engaging with the intervention and stressing the benefits of their involvement. Should a school drop out of the intervention arm of the study prior to completing the peer review process then the remaining partnered school will join another pair to form a trio. Alternatively (and dependent on the stage of the process that has been reached at the time), a WSS Project Director will undertake the review. Should a school drop out of the intervention arm of the study after completing the peer review process, the remaining school will still receive peer support through the regional support network of schools and will be prioritised in the WSS team school visit schedule.

### Relevant concomitant care permitted or prohibited during the trial {11d}

We have not specified any concomitant care to be permitted or prohibited.

### Provisions for post-trial care {30}

Students will be told to tell their teacher if they feel any negative emotions as a result of completing the SDQ (see below) and staff will be made aware of the support required for students in these circumstances. Staff will be advised to look out for signs of distress if students do not talk directly to teachers. Identifying the support structures and services available in a school will be achieved through a school audit prior to the administration of the SDQ. This will ensure that any services and support offered currently in a participating school are clearly identified so that teachers will know who to notify (e.g. parents, other service organisations such as social services) and how to deal with such matters.

The developers (nasen) will oversee the delivery of the SEND Review Process and will take action if necessary to address staff feeling professionally vulnerable. Training and events for SENDCos will provide guidance on how to best manage change.

### Outcomes {12}

Table 1 gives an overview of the primary and secondary outcomes.

**Table 1:** Primary and secondary outcomes

	Outcome	Source	Measure	Analyses
**Primary outcome**				
Attainment	Mark obtained in GSCE English language	Exam boards via schools	Continuous	Pupils with a SEND designation
				All pupils
**Secondary outcomes**				
Attainment	Mark obtained in GCSE mathematics	Exam boards via schools	Continuous	Pupils with a SEND designation
				All pupils
	Grade obtained in GCSE English language	Exam boards via schools	1–9; 0 (unclassified)	Pupils with a SEND designation
	Grade obtained in GCSE mathematics	Exam boards via schools	1–9; 0 (unclassified)	Pupils with a SEND designation
Attendance and exclusions	Unauthorised absences	Schools	Count of number of authorised absences in school year	Pupils with a SEND designation
	Authorised absences	Schools	‘1’ = unauthorised absence observed in the relevant school year, ‘0’ otherwise	Pupils with a SEND designation
	Exclusions	Schools	‘1’ = exclusion observed in the relevant school year, ‘0’ otherwise	Pupils with a SEND designation
Wellbeing	Difficulties	Pupil self-report	Total number of difficulties (max 20)	Pupils with a SEND designation
				All pupils

#### Primary outcome

The primary outcome is attainment in GCSE English language for pupils designated SEND; that is with SEND support or with an Education, Health and Care Plan (ECHP) on Tuesday 1st September 2020. The outcome metric will be standardised (equated) marks in GCSE English Language, obtained via schools from exam boards.

English language was chosen as the primary outcome measure because command of written and spoken language is important in accessing learning in general, and is a determinant of future advancement. Reliance on national examinations for assessment is partly a practical decision but also one that reflects substantive concerns. Importantly, examination scripts are marked ‘blind’ to the pupil’s status with regard to this trial. From a practical perspective, adopting attainment at GCSE as the primary outcome has a number of further advantages. First, considerable resources are devoted by exam boards to the writing and validation of GCSE questions; therefore, examination marks might be considered reliable and valid measures of attainment in and of themselves. Second, the costs of collecting pupil level GCSE results are low compared to the costs of the alternative, which is administering commercial standardised assessment tests. Third, unlike administering separate standardised assessments of literacy and language, using GCSE marks as the primary outcome imposes no additional data collection burden on schools. Fourth, as a measure, it is also less affected by the loss to follow-up than the alternatives.

#### Secondary outcomes

Secondary outcomes are grouped into three categories: (a) further attainment outcomes, (b) attendance and exclusions outcomes and (c) wellbeing outcomes.

##### Further attainment outcomes

The underlying intervention theory provides an account of how the WSS Review is expected to raise general attainment, specifically for SEND pupils but also among the wider student body. As a result, the selection of secondary outcomes is informed by the expectation that aspects of attainment other than English language will improve as a consequence of the WSS Review. Given the importance of attainment in mathematics for future advancement, marks at GCSE mathematics is a secondary outcome for SEND-designated and all pupils respectively.

Whilst our primary outcome focus is on marks (given these might be considered sensitive to small changes in attainment and provide a continuous attainment score), GCSE grades are also of interest and are therefore also included as secondary outcome measures. Grades are well understood—results showing an intervention has an effect on average GCSE grade can be clearly interpreted by stakeholders. Moreover, closing the attainment gap is a central concern and it is a grade that ultimately reflects this and can determine future life prospects (e.g. the opportunity to undertake further study and apply for jobs which specify particular grades as entry requirements).

Grades obtained in both English and mathematics will be on a 1–9 scale with unclassified grades coded to ‘0’.

##### Attendance and exclusions outcomes

The WSS Review aims to bring about a change in school culture, promoting an inclusive and supportive environment as well as addressing specifically the needs of children with SEND in the classroom. These needs can often go unmet leading to poor attendance and in some cases exclusion from school. The trial therefore includes three secondary attendance and exclusion outcomes.

- Authorised absences: a count of the number of authorised absences in the relevant school year depending on the cohort being considered.
- Unauthorised absences: a binary dependent variable for each pupil coded to ‘1’ where an unauthorised absence is observed in the relevant school year, ‘0’ otherwise.
- Exclusions (temporary fixed term, permanent): a binary dependent variable for each pupil capturing whether exclusions from school were recorded in the relevant school years.

##### Wellbeing outcomes

As discussed above, it is anticipated and consistent with the intervention theory of change, that pupil wellbeing will improve as a result of exposure to the WSS Review. Pupil wellbeing is measured using the self-report Strengths and Difficulties Questionnaire (SDQ) for 11–17-year-olds. The SDQ provides a measure of the psychological adjustment of the respondent (or their psychopathology) [11]. The SDQ measure of interest is the ‘total number of difficulties’ score.

#### Baseline measures

Baseline measures will be used for adjusted analyses of trial data for both primary and secondary outcomes. Where possible for each primary and second outcome, a pre-randomisation measure on the same outcome will be obtained in order to form a baseline covariate in the relevant analyses.

##### Attainment

For outcomes in English and Mathematics at GCSE, regardless of whether defined in terms of grade or marks, a baseline covariate will be derived from the raw test scores in Reading and Mathematics at Key Stage 2 (KS2; national tests at 11 years), as appropriate, collected directly from schools before randomisation for each enumerated pupil in years 8 and 9 at September 2020.

##### Attendance and exclusions

Analysis of attendance and exclusions outcomes will be adjusted on the basis of baseline measures of attendance for the school year 2019/20 (noting the shortening of the school year due to the Covid-19 pandemic and the closure of schools). These measures relate to the period of time prior to randomisation, which will take place in June 2021. The covariate will be derived by summing the total number of absences (both authorised and unauthorised) for each pupil for the school year 2019/20.

##### Wellbeing outcomes

An SDQ will be administered to each enumerated pupil at baseline in Years 8 and 9 in May 2021, prior to randomisation. For each pupil that completes the baseline SDQ, we will derive a baseline total difficulties score to be used in the adjusted analysis as a covariate.

##### Additional baseline data items

In addition to the ‘pre-test’ baseline data items mentioned above, further items will be collected at baseline for use in the analysis. These are as follows:

- Date of birth
- Sex
- FSM status
- SEND (ECHP or ‘support’)
- Primary identification of need (e.g. SEND-related need)
- Current class for English (at September 2020)

The analysis discussed below provides for the estimation of effects through (a) an unadjusted analysis, (b) an analysis adjusted for the inclusion of a baseline measure on the dependent variable as a covariate at the pupil level only and (c) full adjusted specification which includes a baseline measure of the dependent variable entered as a covariate at the pupil and school levels as well as further covariates for month of birth, sex and FSM. All specifications will include a region indicator that reflects randomisation by region. Further, the SEND indicator is required in order for the primary analysis to be performed on the subset of the two cohorts years 8 and 9 that are designated either ‘support’ or ‘ECHP’ at the point of randomisation. An FSM indicator is required in order for subgroup analysis to be performed.

### Participant timeline {13}

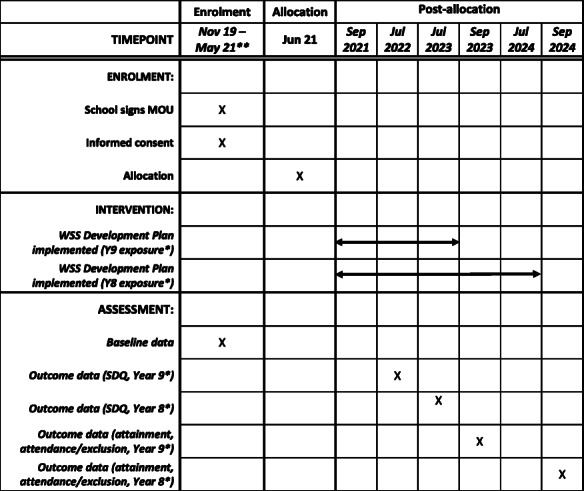



*At September 2020 **Enrolment paused and restarted during this period due to the COVID-19 pandemic

### Sample size {14}

Table 2 sets out the assumptions upon which we have based sample size calculations. It states the relevant minimum detectable effect sizes [12] associated with the primary analysis (SEND designated pupils only), analysis for all pupils (the sample upon which many secondary outcomes will be estimated) and subgroup estimates for pupils ever in receipt of Free School Meals (FSM; the main subgroup analysis).

**Table 2:** Sample size calculations (calculated using PowerUp!^a^)

	All pupils	SEND pupils	FSM pupils
**Minimum Detectable Effect Size (MDES)**	0.19	0.20	0.19
**Pre-test / post-test correlations**			
Level 1 (pupil)	0.70	0.70	0.70
Level 2 (class)	n/a	n/a	n/a
Level 3 (school)	0.32	0.32	0.32
**Intracluster correlations (ICCs)**			
Level 2 (class)	n/a	n/a	n/a
Level 3 (school)	0.20	0.20	0.20
**Alpha**	0.05	0.05	0.05
**Power**	0.80	0.8	0.8
**One-sided or two-sided?**	Two	Two	Two
**Average cluster size**	180	25	42
**Number of schools**			
Intervention	80	80	80
Control	80	80	80
Total	160	160	160
**Number of pupils**			
Intervention	14,400	2000	3360
Control	14,400	2000	3360
Total	28,800	4000	6720

^a^PowerUp! https://www.causalevaluation.org/power-analysis.html

The size of the anticipated sample available for this trial is influenced by the following factors:

- The costs to the developer of working with schools and the available programme budget which determines the maximum possible size of the intervention group.
- The number of schools that the developer could reasonably be expected to recruit in the time available based on our experience of recruiting secondary schools to other similar EEF-funded studies.
- The average size of schools.
- The likely proportions of pupils within schools that are SEND and have ever qualified for free school meals.

In addition, our sample size calculations are based on a number of further assumptions in relation to:

- The correlation between KS2 English raw scores and GCSE English marks.
- The intraclass correlation coefficient at the school level.
- Whether information on the class that individual pupils were in at randomisation for the teaching of English language is available, and if available, is reliable.

In addition to these factors, schools will be assigned to intervention and control groups on a 1:1 basis. Statistical tests will be conducted on the basis of two-sided tests of statistical significance with standard assumptions made regarding types I and II statistical error rates (5 and 20% respectively).

At the outset of the study, the developers informed the research team that they had a budget to work with around 100 schools. This implied the need to recruit some 200 schools to the trial such that subsequent to randomisation 100 schools would be assigned to the intervention. However, previous experience within the research team led to the conclusion that the developers would struggle to recruit 200 schools to this study in the time available. For example, the ‘Evaluating the effectiveness of Eedi formative assessment programme’ study, which also involved the recruitment of mainstream secondary schools to an EEF-funded trial, set out to recruit 180 schools, but despite the best efforts of the developers, only 158 schools agreed to take part in the trial [13]. Given this experience and the fact that the WSS Review will be more demanding on school resources in general, it was felt that 160 schools would be a reasonable but ambitious target that could be achieved by developers in the time available.

With an achieved sample of some 160 schools we can obtain estimates for the average number of pupils in a given focal-year-cohort, the number of pupils that are likely to be SEND as well as the expected number of pupils that had ever qualified for free-school-meals:

- Based on previous studies we expect on average six classes in each year group in mainstream secondary schools and each class will comprise approximately 30 students.
- Thus we expect to find on average about 180 students in each year-group cohort per school (and that there are two cohorts per school).
- Drawing on national publicly available estimates, we expect that around 14% of pupils to be designated SEND [2]. This means that on average we expect to find 25 SEND pupils in each year-based cohort per school
- Nearly a quarter of pupils in maintained secondary schools have qualified for FSM at some point in their school careers [14]. This means we can expect around 42 pupils per cohort, per school to have been in receipt of FSM.

Given these estimates we anticipate that in some schools the numbers of pupils in our sample that will be both SEND and FSM will be very small. For this reason, the sample size estimates above are for all FSM, rather than SEND pupils that are also FSM. Likewise, as set out in the Statistical Methods section we do not propose estimating effects for that subgroup of SEND pupils that are also ever-FSM, due to anticipated small sample sizes.

We obtained an estimate of the correlation between KS2 Raw score for English and GCSE English language attainment from the analysis provided by the Education Endowment Foundation [15]. The assumption used for the intraclass correlation coefficient is 0.20 (proportion of the total variance at the school level), and though possibly conservative is the assumption used for many EEF-funded studies with GCSE attainment as a primary outcome.

Taken together these assumptions and other information lead to estimated minimum detectable effect sizes for the primary analysis of 0.20 of a standardised mean difference, and 0.19 for samples based on all pupils and those ever-FSM respectively. Given the prospects for school recruitment, the time frame over which recruitment needed to take place and the available budget, assuming 80% power, these effects are the smallest true effects that would lead to results reaching levels of statistical significance at the 95% level. If we assume a standard deviation of around 50–60 marks in GCSE English language, an effect size of 0.20 translates, very approximately, into an average improvement among SEND students in intervention over control schools of around 10–12 Marks.

### Recruitment {15}

We initially identified that it would be necessary to recruit schools that had not previously engaged with WSS Review, thus requiring a substantial effort on behalf of the developer who was primarily responsible for recruitment. The developer was however in a strong position to do this, having a national reach and relationships with schools across all regions of England. Recruitment was supported by the trial sponsor (EEF) who have a similar national profile and established channels to aid the recruitment of schools for research. Stratification by region has allowed for efficiencies in recruitment thus far and will prevent unnecessary delays and achieve balance in the number of intervention and control schools in each region.

## Assignment of interventions: Allocation

### Sequence generation {16a}

Randomisation is stratified and will be performed regionally in five batches. Based on the sample size calculations, the developers aimed to recruit a minimum of 32 state secondary schools in each region, more if possible (although for the purposes of pairing an even number of schools is required in each region).

The randomisation process is the same for each region:

- The authors will assign each recruited school a random number drawn from a uniform distribution in STATA v16 (a random number seed will be set and stored so that it can be retrieved at a later date).
- Schools will be ordered by the uniform random number on an ascending basis.
- Two groups of schools will be formed by splitting the ordered list of schools in half—the first group will be group 1, and the second group 0.
- Group 1 will be assigned to the treatment condition and group 0 will be assigned to the control condition.

### Concealment mechanism {16b}

Allocation concealment will be achieved as randomisation will be conducted by computer, with school identifiers unknown to the research statistician running the randomisation script. Assignment status will be made known to all enlisted schools in June 2021.

### Implementation {16c}

The researchers will assign schools to the intervention by the procedure outlined above (sequence generation).

## Assignment of interventions: Blinding

### Who will be blinded {17a}

There are no specific measures for blinding. However, whilst the group assignments will be known to the researchers, data collectors, developers, SENDCos and other school staff, it is unlikely that pupils will be aware of the implementation of the intervention as distinct from other school activities.

### Procedure for unblinding if needed {17b}

The design is open label so unblinding will not occur.

## Data collection and management

### Plans for assessment and collection of outcomes {18a}

All data outcome and baseline data collection is being undertaken by FFT, a non-profit organisation “focussed on providing accurate and insightful information to schools which enables pupils to achieve their full potential and schools to improve” [16]. Many schools in the UK use FFT’s Aspire reporting and data tool, and FFT therefore have direct access to the majority of data to be collected in the trial. Where a trial school uses Aspire, data will be sent to the school by FFT for validation before being returned to FFT by the school. For schools that do not use Aspire, FFT will directly request the data from the school. FFT have well-established internal processes for the quality assurance of data, and a second level of checks will be implemented by the research team.

#### Outcome data

In order to obtain attainment data, schools in the trial will be approached by FFT and asked for the marks and grades obtained by individual students at GCSE and provided to the school by exam boards. Data will be obtained at September 2023 (for pupils in year 9 at September 2020), and September 2024 (for pupils in year 8 at September 2020). The measures of Grade achieved by pupils will be equivalent to those available through the National Pupil Database (NPD).

At the point GCSE Grades are extracted from school data systems by FFT, absence and exclusion data will be obtained for pupils for the school year 2022/2023 for year 9 pupils (at September 2020) and the school year 2023/2024 for year 8 pupils (at September 2020).

FFT will also co-ordinate administration and data collection of wellbeing data, measured using the self-reported Strengths and Difficulties Questionnaire (SDQ) for 11–17-year-olds. The SDQ contains 25 items, 20 of which form four sub-scales: emotional symptoms, conduct problems, hyperactivity/inattention and peer problems. A score on each sub-scale is obtained and then the total number of difficulties derived from summing across the subscales. The additional five items form a separate prosocial behaviour scale which we are not intending to use in our analysis. The validity and reliability of the SDQ are discussed in Goodman & Goodman [17, 18].

Prior to randomisation (in June 2021), the SDQ will be administered online to the enumerated sample of pupils (overseen by teachers and teaching assistants) in both years 8 and 9 at baseline. The SDQ will again be administered at June/July 2022 (for year 9s) and June/July 2023 (for year 8s). The choice of timing of the follow-up SDQ measurements was informed by the need to avoid administering the instrument in year 11, when there are significant calls on teachers’ time and school resources in general. Doing so also provides the possibility of using well-being as a mediating variable in analyses of attainment [19], thereby taking into account the required temporal ordering of measurements to permit this.

#### Baseline data

FFT will also collect baseline data from Aspire and non-Aspire schools as the basis for adjusted analyses of trial data for both primary and secondary outcomes. Where possible for each primary and secondary outcome, a pre-randomisation measure on the same outcome will be obtained in order to form a baseline covariate in the relevant analyses.

This includes the following:

- Raw test scores in Reading and Mathematics at KS2 for each enumerated pupil in Years 8 and 9 at September 2020.
- Absence and exclusion data for each pupil for the school year 2019/2020.
- Additional baseline data items (date of birth, sex, FSM status, SEND (ECHP or support), Primary identification of need, current class for English (at September 2020).

##### Linking records for primary, secondary and baseline measurements

Baseline data will be linked together to form the trial database using full name, date of birth, school Unique Reference Number (URN) and Unique Pupil Number (UPN). As stated, SDQs are administered separately at baseline (May 2021) and at a future point in time. Pupil level records generated from the SDQs will be linked to the baseline data records using UPN, full name and date of birth. Outcome data will also be linked accordingly.

### Plans to promote participant retention and complete follow-up {18b}

The researchers have started working with the developers to implement a communication strategy which emphasises to schools the importance of adhering to the trial. This was clearly communicated in the Memorandum of Understanding, to which all participating schools agreed. There is, however, a high chance that some schools recruited to the trial decide to withdraw, and this sample loss might both reduce the precision of statistical estimates and introduce bias. Where attrition occurs, steps will be taken in the analysis to test various assumptions regarding missingness and to assess consequences for bias and precision. Given the researchers’ experience of running other educational trials, we are not expecting a significant number of pupils to subsequently withdraw after enrolment is completed.

### Data management {19}

The Memorandum of Understanding (MOU) for schools and Data Sharing Agreement clearly specify the responsibilities of all parties with regard to the supply and processing of data. The process of data collection and quality assurance is described in the ‘Plans for assessment and collection of outcomes {18a}’ section above.

### Confidentiality {27}

Data will be transferred to the researchers via FFT’s secure transfer site. All research data will be held within a secure research area agreed with the University’s Head of Information Security. The information collected will be used for research purposes only and no information that can identify individuals will be used for any other purpose. Any personal data collected and held by Manchester Metropolitan University, nasen and FFT will be destroyed in accordance with the GDPR when it is no longer required, and no later than July 2025.

Any information identifying students will be given a unique ‘meaningless identifier’ prior to analysis in order to reduce risk. Data deposited in the EEF data archive (in the ONS SRS) will also include Pupil Matching References for any subsequent longitudinal matching which is not part of this trial. Therefore, data will only be released subsequently to ONS Accredited Researchers in an anonymised format.

### Plans for collection, laboratory evaluation and storage of biological specimens for genetic or molecular analysis in this trial/future use {33}

See Item 26b—there will be no biological specimens collected.

## Statistical methods

### Statistical methods for primary and secondary outcomes {20a}

Both primary and secondary analysis will follow the intention to treat principle.

#### Primary analysis

Focusing first on the primary analysis, statistical estimates of the effect of exposure on marks at GCSE English will be obtained from a hierarchical linear model (the estimator), in which pupils are clustered within schools. This model will be fitted to data for SEND pupils only. Three regression model specifications are proposed, where the standardised or equated mark for each pupil is the dependent variable^1^, and the regression models include the following covariates:

- *Model 1*: binary intervention group indicator coded to ‘1’ if the school is assigned to the intervention ‘0’ otherwise, plus regional fixed effects (representing strata).
- *Model 2*: As above, with KS2 Reading raw score as a covariate expressed as a departure from the school mean for each pupil at the pupil level, and as a school average departure from the overall mean at the school level
- *Model 3*: As model 2, with additional covariates representing sex, month of birth, unauthorised absences in the year prior to randomisation and FSM variables.

The effect size, consistent with Hedges’ *g*, will be obtained from model 2, as set out in EEF guidance [20]. The effect size parameter is written:

$$ ES=\frac{\mu^T-{\mu}^c}{\sqrt{\sigma_i^2+{\sigma}_j^2}} $$

A sample estimate of *μ*^*T*^ − *μ*^*c*^, where *μ*^*T*^ is the mean of the outcome in the treatment group and *μ*^*c*^ the mean in the control group, is derived from the coefficient obtained on the binary intervention group indicator from model 2 above. The denominator ${\sigma}_i^2+{\sigma}_j^2$, where *i* indexes for the school and *j* the pupil, are the variances at the school and pupil levels respectively, such that the intraclass correlation coefficient is ${\sigma}_i^2/{\sigma}_i^2+{\sigma}_j^2$. A sample estimate for the denominator is obtained from the total unconstrained pooled variance as described in Hedges [20], who also provides an equation for the variance of the sample estimate for the effect size. Uncertainty will be assessed through computation of 95% confidence intervals and *p* values.

#### Secondary analysis

The secondary analysis will involve estimation of effects on a range of outcomes discussed previously for the full year-group cohort samples (years 8 and 9) and for SEND pupils only (years 8 and 9).

Table 3 sets out the secondary analysis to be conducted on the full cohort samples. The analysis for years 8 and 9 will appear in separate reports. Hypothesis tests for the treatment effects in each specification will be reported in the form of *p* values and 95% confidence intervals. For the secondary analysis, treatment effect estimates based on continuous outcomes will be reported as effect sizes (Hedges’ *g*), where outcomes are binary as relative risk ratios and for count outcomes as incident rate ratios. Table 4 sets out the secondary analysis to be performed on the SEND only subsamples.

**Table 3:** Secondary analysis—model specifications—full cohort samples years 8 and 9 cohorts (as at September 2020)

Dependent variable	Model	Intervention group indicator	Strata indicator	Covariates	Cohort (at 09/20)
**Attainment outcomes**					
**GCSE English language Mark (standardised)**	Linear hierarchical	Yes	Yes	• KS2 Reading raw score at pupil and school levels • Month of birth • Sex • FSM	Year 9
**GCSE English language Mark (standardised)**	Linear hierarchical	Yes	Yes	• KS2 Reading raw score at pupil and school levels • Month of birth • Sex • FSM	Year 8
**GCSE Mathematics Mark (standardised)**	Linear hierarchical	Yes	Yes	• KS2 mathematics raw score at pupil and school levels • Month of birth • Sex • FSM	Year 9
**GCSE Mathematics Mark (standardised)**	Linear hierarchical	Yes	Yes	• KS2 mathematics raw score at pupil and school levels • Month of birth • Sex • FSM	Year 8
**Wellbeing outcomes**					
**Total difficulties (SDQ)**	Linear hierarchical	Yes	Yes	• Total difficulties baseline score • Month of birth • Sex • FSM	Year 9
**Total difficulties (SDQ)**	Linear hierarchical	Yes	Yes	• Total difficulties baseline score • Month of birth • Sex • FSM	Year 8

**Table 4:** Secondary analysis—model specifications—SEND only samples years 8 and 9 cohorts (as at September 2020)

Dependent variable	Model	Intervention group indicator	Strata indicator	Covariates	Cohort (at 09/20)
**Attainment outcomes**					
**GCSE Mathematics Mark (standardised)**	Linear hierarchical	Yes	Yes	• KS2 mathematics raw score at pupil and school levels • Month of birth • Sex	Year 9
**GCSE Mathematics Mark (standardised)**	Linear hierarchical	Yes	Yes	• KS2 mathematics raw score at pupil and school levels • Month of birth • Sex	Year 8
**GCSE English Language Grade 1-9**	Linear hierarchical	Yes	Yes	• KS2 reading raw score at pupil and school levels • Month of birth • Sex	Year 9
**GCSE English Language Grade 1-9**	Linear hierarchical	Yes	Yes	• KS2 reading raw score at pupil and school levels • Month of birth • Sex	Year 8
**GCSE Mathematics Grade 1-9**	Linear hierarchical	Yes	Yes	• KS2 mathematics raw score at pupil and school levels • Month of birth • Sex	Year 9
**GCSE Mathematics Grade 1-9**	Linear hierarchical	Yes	Yes	• KS2 mathematics raw score at pupil and school levels • Month of birth • Sex	Year 8
**Attendance and exclusion outcomes**					
**Number of authorised absences in previous school year 2022/2023**	Count negative binomial hierarchical	Yes	Yes	• Number of authorised absences in school year 2019/2020 • Month of birth • Sex	Year 9
**Number of authorised absences in previous school year 2023/2024**	Count negative binomial hierarchical	Yes	Yes	• Number of authorised absences in school year 2019/2020 • Month of birth • Sex	Year 8
**At least one unauthorised absence in school year 2022/2023**	Binary logistic hierarchical	Yes	Yes	• Number of absences in school year 2019/2020 • Month of birth • Sex	Year 9
**At least one unauthorised absence in school year 2023/2024**	Binary logistic hierarchical	Yes	Yes	• Number of absences in school year 2019/2020 • Month of birth • Sex	Year 8
**At least one exclusion from school in school year 2022/2023**	Binary logistic hierarchical	Yes	Yes	• Number of absences in school year 2019/2020 • Month of birth • Sex	Year 9
**At least one exclusion from school in school year 2023/2024**	Binary logistic hierarchical	Yes	Yes	• Number of absences in school year 2019/2020 • Month of birth • Sex	Year 8
**Wellbeing outcomes**					
**Total difficulties (SDQ)**	Linear hierarchical	Yes	Yes	• Total difficulties baseline score • Month of birth • Sex	Year 9
**Total difficulties (SDQ)**	Linear hierarchical	Yes	Yes	• Total difficulties baseline score • Month of birth • Sex	Year 8

### Interim analyses {21b}

There are two cohorts of pupils that are the focus of this trial; those pupils in intervention and control schools in years 8 and 9 at September 2020. Following training, it is anticipated that school Development Plans will reflect WSS processes and actions from September 2021. Longitudinally the year 9 cohort would capture the near-term effects of the intervention at GCSE, and the Year 8 cohort would capture the more medium-term effects.

Analysis of outcomes for the year 8 cohort will only proceed if acceptable implementation fidelity is achieved (determined by the IPE—see the ‘Discussion’ section). The results of the main phase of the research, focusing on students in year 9 as of September 2020, will be published in a report in January 2024. If the data from the main phase are robust, then the results of the additional phase of the research, focusing on students in year 8 as of September 2020, will be published in a report in February 2025.

### Methods for additional analyses (e.g. subgroup analyses) {20b}

Consistent with EEF’s analysis requirements [21], we will run a specification similar to that for the primary analysis, where the FSM indicator is interacted with the intervention group indicator variable and separately on the ever-FSM subsample. This will therefore be a full adjusted specification which includes a baseline measure of the dependent variable entered as a covariate at the pupil and school levels as well as further covariates for month of birth, sex and FSM.

### Methods in analysis to handle protocol non-adherence and any statistical methods to handle missing data {20c}

In order to perform instrumental variables regression and recover estimates of treatment effects, the point at which a unit is understood to have complied with their assignment needs to be determined. Given that the intervention is a whole school programme, pupils’ exposure is determined by school compliance. In order to use instrumental variables, we must also be sure that among other things, pupils in schools we declare to be non-compliant (among intervention schools) are not affected in any way by the intervention.

A conservative, minimal and therefore strict definition of compliance would be that schools assigned to the intervention that do not take up the 1-day SEND Reviewer training are non-compliant. Likewise, schools assigned to control that take-up training are also non-compliant. The only other source of exposure to WSS, outside the control of the developers, that a school assigned to control or intervention groups might be subject to, is downloading the intervention brochure/guidance from the developer website. In order to prevent this, the developer has removed the guidance and taken down the link for the duration of the study. Therefore we proceed on the basis that in order for an intervention school to be minimally compliant they must have received training. Likewise for a control school, they must receive no training. We propose to consult the developers’ records of training received in order to extract a measure of compliance.

There is a high chance that some schools recruited to the trial decide to withdraw, and this sample loss might both reduce precision of statistical estimates and introduce bias. Drawing on our experience and that of the developer, we will devise a strategy to limit school level attrition. Where attrition occurs, we will take steps during analysis to test various assumptions regarding missingness and assess to consequences for bias and precision. Other sources of missingness can result from misrecording of identifying data for pupils. This can be mitigated through carrying out extensive checks on students records at randomisation (see the ‘Plans for assessment and collection of outcomes {18a}’ section above). Where appropriate to do so, an assumption of missing at random will be explored through sensitivity analysis implementing multiple imputation.

### Plans to give access to the full protocol, participant level-data and statistical code {31c}

The full protocol was revised due to changes stemming from school closures in response to the COVID-19 pandemic. Version 2.0 (published on 30th October 2020) is available online^22^.

On completion of the trial, the participant level dataset will be made available within the EEF data archive within the ONS SRS. As per the EEF’s protocol, this will be done by FFT.

## Oversight and monitoring

### Composition of the coordinating centre and trial steering committee {5d}

#### Principal Investigators (Professors Steve Morris and Cathy Lewin)

- Design and conduct of trial
- Preparation of protocol and revisions; agreement of final protocol
- Organising regular meetings between research team, developer and trial sponsor
- Publication of study reports
- Completing ethics committee applications
- Budget administration

#### Research team (protocol authors)

- Study planning
- Responsible for maintenance of trial master file (baseline, SDQ and outcome data)
- Quantitative data verification
- Randomisation
- Quantitative data analysis
- IPE data collection and analysis

#### Developers (nasen)

- Recruitment of schools and ongoing school liaison
- Implementation of WSS Review process, including initial training

#### FFT

Collection, verification and preparation of baseline, SDQ and outcome data

### Composition of the data monitoring committee, its role and reporting structure {21a}

As the trial has been assessed to pose a minimal risk to pupils, a formal data monitoring committee is not required. However, the researchers will undertake review data on an ongoing basis as the trial progresses (particularly with regard to SDQ data) to monitor for any apparent adverse outcomes.

### Adverse event reporting and harms {22}

The developers will oversee the delivery of the WSS Review Process and will take action if necessary to address school staff feeling professionally vulnerable. Schools will monitor for any harms in pupils who have completed the SDQ and will address them as per their established pastoral care processes.

### Frequency and plans for auditing trial conduct {23}

The researchers, sponsor and developer will meet regularly (bi-weekly during the initial phase) to monitor the progress and conduct of the trial.

### Plans for communicating important protocol amendments to relevant parties (e.g. trial participants, ethical committees) {25}

Protocol amendments will be communicated as and if required by the developer, who will have close working relationships with trial schools.

### Dissemination plans {31a}

The results of the main phase of the research, focusing on students in year 9 as of September 2020, will be published in a report in January 2024. If the data from the main phase are judged to be reliable, then the results of the additional phase of the research, focusing on students in year 8 as of September 2020, will be published in a report in February 2025. The evaluation team may publish articles in academic journals once the main reports have been published. All participants and schools will be fully anonymised in any reporting. In addition, we will produce a short report of findings from the SDQ data for each school in the trial in order to encourage their engagement and achieve a high response rate. Schools will receive these reports at the end of the trial after GCSE examinations.

## Discussion

The proposed trial has undergone a number of minor changes during initial recruitment. The trial timetable was altered on account of disruption to schools caused by the COVID-19 pandemic (the majority of English schools were closed between March and September 2020, although pupils received online teaching). The full online protocol [10] therefore reflects changes which were necessary to the original plan, and this document includes further minor timeline alterations (beyond those in the full online protocol), necessary due to ongoing disruption caused by the pandemic.

In addition to the details pertaining to the trial structure and effect size estimation provided in other parts of this document, the research also considers implementation factors: understanding the intervention theory of change, an implementation and process evaluation (IPE) and a cost evaluation. Brief details of these are included in this protocol and full accounts can be found in the full online protocol [10].

## Theory of change

**Fig. 1:**
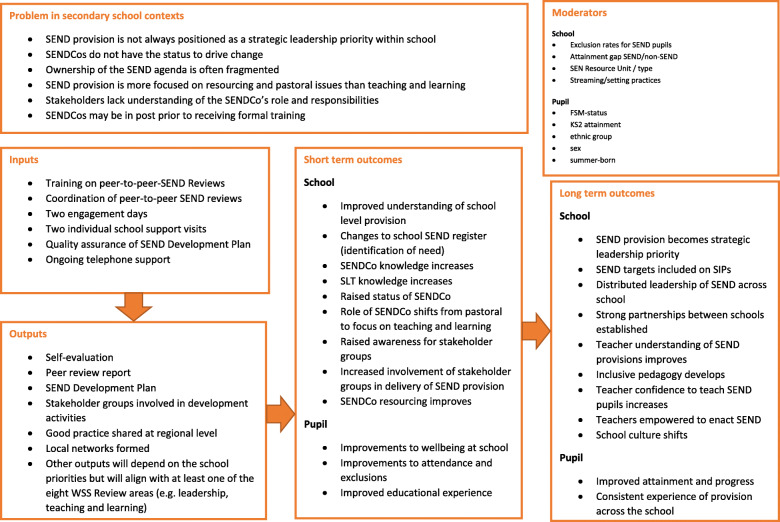
WSS Review Logic Model

Figure 1 describes the WSS Review logic model. It captures core inputs, outputs (in terms of what will be produced or happen as a result of the process), short-term outcomes at both the school level and the pupil level and the long-term outcomes.

The short-term outcomes are effective mediators of the causal impact on students. These are the changes that are hypothesised to be necessary in order for student experience to improve, for their sense of well-being to increase, and for them to be more engaged in learning. As a consequence, it is anticipated that absenteeism, as well as fixed-term and permanent exclusions, will be reduced. In turn, it is expected that this will lead to longer-term cultural shifts and ultimately to improvements in students’ attainment and progress. The model was developed initially by the WSS delivery team (nasen) and revised following a workshop with the researcher in September 2019.

### Implementation and process evaluation

The IPE will focus on implementation delivery (e.g. engagement with the review process and follow-up support from nasen, implementation of Development Plans, changes to policy and practice) and, for comparison, what takes place in relation to SEND provision in control schools. Table 5 outlines the data collection methods upon which the IPE will be based.

**Table 5:** Overview of IPE methods

Research methods	Data collection methods	Participants/ data sources (type, number)	Data analysis methods	Research questions addressed [10]	Implementation/ logic model relevance
**Survey (pre/post-test)**	SLT/SENDCo online survey	SLT/SENDCo (160)	Descriptive Inferential Cross-tabulations Mixed coding Thematic analysis	RQ1 RQ2 RQ3 RQ4	Moderators Usual practice Context Cost
	Telephone semi-structured interviews	10 control group SENDCos	Description—pen portraits	RQ2	Usual practice
**Observations**	Observation of WSS regional training	3 (of 5) events		RQ3	Compliance Activities Fidelity Quality
	Observation of WSS regional engagement days 1 and 2	5 events × 2		RQ1 RQ3	Activities Fidelity Quality
**Case studies (5; case study unit = pair of secondary schools; analytical approach = methodological and participant triangulation)**	Document analysis	SIP SEND information report SEND policy LA local offer WSS Review SEND Development Plan	Mixed coding Thematic analysis Cross-case analysis	RQ1 RQ3	Context Quality Moderators
	Observation of WSS support visit	10 case study schools	Mixed coding Thematic analysis Cross-case analysis	RQ1 RQ3	Activities Fidelity Quality
	Interviews with key stakeholders in case study schools	Depends on focus of school action plan. 5 interviews × 3 visits	Mixed coding Thematic analysis Cross-case analysis	RQ1 RQ3 RQ4	Fidelity Cost Context Moderators Quality Reach Responsiveness Programme Differentiation
	Observation of activities relating to delivery of action plan	Depends on focus of action plan. Maximum of 2 observations × 3 visits	Mixed coding Thematic analysis Cross-case analysis	RQ1 RQ3	Activities Context Quality Reach Responsiveness
	Stakeholder groups surveys	Depends on focus of action plan. Maximum of 3 surveys × 2 administrations	Descriptive Cross-tabulations	RQ1	Context Moderators Quality Reach Responsiveness
**Semi-structured interviews**	Telephone interviews with WSS staff	5 key staff from WSS	Mixed coding Thematic analysis	RQ1 RQ3 RQ4	Context Cost Fidelity

The IPE will be underpinned by the theory of change, investigating implementation dimensions and influential factors. Particular attention will be paid to the diversity in action plans, the reach and uptake of proposed developments, any adaptations that take place during implementation and costs of delivery (fixed and variable). We intend to consider fidelity in depth in selected case study schools. This will include ascertaining levels of engagement with the WSS Review Process steps and activities, and involvement of the school Senior Leadership Team (SLT) and governors.

The IPE considers the following questions:

1. How is the WSS Review process implemented in secondary school contexts?
  - What are the areas of focus that schools prioritise and how are these understood by stakeholders?
  - What initiatives and/or actions are taken by stakeholders in response to the WSS process?
  - What levels of support do SENDCos require and from whom?
  - What are the strengths and challenges of the WSS Review process, e.g. pairing, networking, training?
  - How do different stakeholder groups (e.g. students, teachers, governors) experience the WSS Review process and how does it impact on them?
  - What factors contribute to the SEND Review process being effective (or not)?
2. What comparable initiatives and/or actions are taken within control group schools?
  - What is the initial position?
  - How does this change over time?
3. How was the WSS Review process delivered and supported in relation to compliance, fidelity, quality, reach, responsiveness and programme differentiation?
  - What is the reach in terms of the involvement of departments, staff members (from senior leaders to teaching assistants), governors and other stakeholders such as parents?
  - What is the responsiveness in terms of how each of the stakeholder groups involved engages with the outcomes of the WSS Review process?
  - What is the programme differentiation in relation to how the outcomes of the WSS Review process differ from prior SEND and inclusion practices in the intervention schools?

### Cost evaluation

The cost of programme delivery will be estimated using the principles set out by the EEF. The primary question to be addressed is: what is the per pupil cost of the intervention? Data collection for this estimation take place alongside that of the IPE and the estimate will be subject to sensitivity analysis in order to address heterogeneity between schools and uncertainty about the value of resources. Key methods of gathering this information will therefore be the surveys of SENDCos (baseline and at follow-up in 2022 and 2023), telephone interviews with SENDCos, and face-to-face interviews with key stakeholder groups. In addition, financial documents pertaining to the intervention will be sought where appropriate (e.g. from nasen, schools) and will be analysed in order to triangulate findings from the surveys and interviews.

## Trial status

Protocol version 2.0 (30th October 2020). Revised protocol [10] accounting for changes stemming from school closures in response to COVID-19 pandemic (timelines further subject to minor amendments in this document). Trial recruitment ended 1st February 2021.

## Data Availability

The final trial dataset will be made available in the EEF’s data archive within the Office for National Statistics’ Secure Research Service. It will therefore be publically available to any individual who is an ONS Accredited Researcher.

## Abbreviations

DfE: Department for Education
ECHP: Education, Health and Care Plan
EEF: Education Endowment Foundation (the trial sponsor and funder)
FSM: Free School Meals (FSM eligibility is used as a proxy for deprivation)
GCSE: General Certificate of Standard Education (tests usually sat at 16 years)
IPE: Implementation Process Evaluation
KS2: Key Stage 2 (7–11 years)
LA: Local Authority
LAC: Looked After Children
MAT: Multi-academy Trust
MoU: Memorandum of Understanding
Nasen: National Association for Special Educational Needs (the developer of the WSS Review)
NPD: National Pupil Database
ONS: Office for National Statistics
SDQ: Strengths and Difficulties Questionnaire
SEND: Special Educational Needs and Disabilities
SENDCo: Special Educational Needs Co-ordinator
SIP: School Improvement Plan
SLT: Senior Leadership Team (in schools)
SRS: Secure Research Service
WSS: Whole School SEND
UPN: Unique Pupil Number
URN: Unique Reference Number (school)

## Footnotes

1 Given that the primary analysis will be performed on the subset of the sample designated SEND at September 2020, the sample observations on marks and grades may not be normally distributed and/or sample data might contain extreme values. The approach to tackling this problem will depend on the characteristics of the resulting distributions. For example, if a small number of pupils record high marks causing the distribution of marks to be left skewed then the marks might be log transformed, and sensitivity analysis performed with the log transformed measure as the dependent variable. Alternatively, sensitivity analysis might be performed on a transformed dependent variable that is trimmed of extreme values; for example, 5% of the sample at the extremes might be dropped for such an analysis. Finally, we do not anticipate floor effects in the data, but if these are encountered sensitivity analysis can be conducted using a maximum likelihood Tobit regression model.
